# Exploitation of Novel *Bt* ICPs for the Management of *Helicoverpa armigera* (Hübner) in Cotton (*Gossypium hirsutum* L.): A Transgenic Approach

**DOI:** 10.3389/fmicb.2021.661212

**Published:** 2021-04-29

**Authors:** Kesiraju Karthik, Jyotsana Negi, Maniraj Rathinam, Navinder Saini, Rohini Sreevathsa

**Affiliations:** ^1^ICAR-National Institute for Plant Biotechnology, New Delhi, India; ^2^Division of Genetics, ICAR-Indian Agricultural Research Institute, New Delhi, India

**Keywords:** *Bacillus thuringiensis*, chimeric proteins, *cry1AcF*, *cry2Aa*, domain swapping, transgenic cotton, *in planta* transformation

## Abstract

Cotton is a commercial crop of global importance. The major threat challenging the productivity in cotton has been the lepidopteron insect pest *Helicoverpa armigera* or cotton bollworm which voraciously feeds on various plant parts. Biotechnological interventions to manage this herbivore have been a universally inevitable option. The advent of plant genetic engineering and exploitation of *Bacillus thuringiensis* (*Bt*) insecticidal crystal proteins (ICPs) marked the beginning of plant protection in cotton through transgenic technology. Despite phenomenal success and widespread acceptance, the fear of resistance development in insects has been a perennial concern. To address this issue, alternate strategies like introgression of a combination of cry protein genes and protein-engineered chimeric toxin genes came into practice. The utility of chimeric toxins produced by domain swapping, rearrangement of domains, and other strategies aid in toxins emerging with broad spectrum efficacy that facilitate the avoidance of resistance in insects toward cry toxins. The present study demonstrates the utility of two *Bt* ICPs, *cry1AcF* (produced by domain swapping) and *cry2Aa* (produced by codon modification) in transgenic cotton for the mitigation of *H. armigera.* Transgenics were developed in cotton cv. Pusa 8–6 by the exploitation of an apical meristem-targeted *in planta* transformation protocol. Stringent trait efficacy-based selective screening of T_1_ and T_2_ generation transgenic plants enabled the identification of plants resistant to *H. armigera* upon deliberate challenging. Evaluation of shortlisted events in T_3_ generation identified a total of nine superior transgenic events with both the genes (six with *cry1AcF* and three with *cry2Aa*). The transgenic plants depicted 80–100% larval mortality of *H. armigera* and 10–30% leaf damage. Molecular characterization of the shortlisted transgenics demonstrated stable integration, inheritance and expression of transgenes. The study is the first of its kind to utilise a non-tissue culture-based transformation strategy for the development of stable transgenics in cotton harbouring two novel genes, *cry1AcF* and *cry2Aa* for insect resistance. The identified transgenic events can be potential options toward the exploitation of unique *cry* genes for the management of the polyphagous insect pest *H. armigera*.

## Introduction

Cotton is an economically important crop grown worldwide in about 80 countries and planted in an average area of 329.49 MH. In India, during 2019–2020, cotton was planted in 125.84 million hectares yielding a produce of 360 million bales with 486 kg/ha productivity. However, yield per hectare is still lower than the world average of about 762 kg/ha^1^. Despite the use of more cultivable land for cotton cultivation, per hectare yield is low, possibly due to the low availability of seeds conferring traits for insect pest resistance. Together with numerous lepidopteran insect pests that attack the crop, cotton bollworm *Helicoverpa armigera*, a polyphagous insect pest has always been a serious concern.

Biotechnological involvement and exploitation for the management of insects as devastating as the boll worm have been phenomenal. Genetic engineering for the development of insect resistant plants has been considered as a revolutionary contribution in agricultural biotechnology. Transgenesis in economically important crop plants like cotton (Chen et al., 2017; Ribeiro et al., 2017; Siddiqui et al., 2019; Bajwa et al., 2020; Jadhav et al., 2020; Katta et al., 2020); soybean (Marques et al., 2017; Moghaieb et al., 2019; Qin et al., 2019; Bacalhau et al., 2020); maize (Moscardini et al., 2020); pigeon pea (Ghosh et al., 2017; Singh et al., 2018; Ramkumar et al., 2020); chick pea (Das et al., 2017); cow pea (Bett et al., 2017; Addae et al., 2020; Kumar et al., 2021); sweet potato (Zhong et al., 2019); jute (Majumder et al., 2020); castor (Muddanuru et al., 2019), etc., by the introgression of insecticidal proteins encoding genes from *Bacillus thuringiensis* (*Bt*) resulted in plants with improved ability to mitigate the pest load. Reduced topical applications of chemical pesticides and increased agricultural productivity have been major achievements of transgenic technology (Fleming et al., 2018) in crop improvement. However, narrow range of specificity toward insect pests and the fear of resistance development have been crucial concerns (Tabashnik et al., 2013; Tabashnik and Carrière, 2017, 2019).

Consequently began a new era of gene pyramiding (c*ry1Ac* and c*ry2Ab* in Bollgard II, Monsanto) for insect resistance which involved the introduction of gene combinations to provide broad spectrum of efficacy toward insect herbivory (Chen et al., 2017; Moghaieb et al., 2019; Siddiqui et al., 2019; Katta et al., 2020; Muralimohan et al., 2020). Pyramided genes exhibited effective toxicity and also restricted evolution of resistant pest population in engineered commercial crop plants (Razaq et al., 2019; Wei et al., 2019).

Alternatively, to circumvent resistance, several novel strategies like gene pyramiding and generation of chimeric toxins (Zghal et al., 2017; Khabbazi et al., 2018; Sellami et al., 2018; Rathinam et al., 2019; Zubair et al., 2019; Salim et al., 2020); RNAi-mediated knockdown (Tian et al., 2015; Han et al., 2017; Luo et al., 2017; Ni et al., 2017; Shen et al., 2017); CRISPR/Cas9 mediated genome editing (Zhang et al., 2018; Bisht et al., 2019; Tyagi et al., 2020) and multiomic approaches (Du et al., 2018; Peng et al., 2020) for the management of insect resistance have also become an integral part of pest management through biotechnology.

The use of synthetic or protein-engineered chimeric toxins as one of the resistance management strategies has likewise been considered as a viable approach. Protein-engineered chimeric toxins produced by domain swapping, rearrangement of domains and other strategies aided toward broad spectrum efficacy and avoidance of resistance in insects toward cry toxins (Shah et al., 2017; Zghal et al., 2017; Sellami et al., 2018; Rathinam et al., 2019; Baum et al., 2020). Structural similarity of different insecticidal crystal proteins (ICPs) with well-predictable roles for each of the three cry toxin domains has fruitfully allowed application of domain swapping for the generation of hybrid *Bt* toxins with comprehensive resilience and novel specificities (Rathinam et al., 2019; Baum et al., 2020).

A 26-fold increase in toxicity was demonstrated by the combination of *cry1Ac* and *cry1F* in a 1:1 ratio, suggesting a synergistic effect against *H. armigera*. Accordingly, *cry1AcF* (Indian Patent number: 237912), a chimeric *cry* gene was developed by the fusion of domains I and II from *cry1Ac* and domain III from *cry1F* (Ramu et al., 2012; Rathinam et al., 2019). Similarly, *cry2Aa* (GenBank accession ID: ABW87832.1), is known to exhibit broad spectrum efficacy against two insect orders (Singh et al., 2018; Kumar et al., 2021), lepidoptera and diptera (Qiu et al., 2017; Zhao et al., 2017; Goje et al., 2020). Efficacy of these genes has been effectively demonstrated against *H. armigera* in both model and crop plants (Muralimohan et al., 2020; Ramkumar et al., 2020; Kumar et al., 2021).

In the present study, we demonstrate the utility of two *Bt* ICPs, *cry1AcF*, and *cry2Aa* for the management of *H. armigera* in transgenic cotton. Considering the recalcitrant nature of cotton to regeneration by tissue culture, utilization of an apical meristem targeted *in planta* transformation for the development of stable events is an added advantage (Kesiraju and Sreevathsa, 2017; Karthik et al., 2020a; Kesiraju et al., 2020). Our results reconfirmed both the amenability of cotton to *in planta* transformation strategy as well as efficacy of the two cry toxin genes toward the management of the devastating cotton bollworm, *H. armigera*.

## Materials and Methods

### *In planta* Transformation of Cotton

Transformants in cotton were developed following the apical meristem-targeted *in planta* transformation strategy in the cv. Pusa 8–6. Binary vector pBinAR, independently harbouring *Bt* genes *cry1AcF* (Ramu et al., 2012) and *cry2Aa* (Singh et al., 2018; synthesized, cloned and validated at ICAR-National Institute for Plant Biotechnology, New Delhi, India), driven by CaMV 35S promoter in *Agrobacterium tumefaciens* strain EHA105 were used for transformation. Neomycin phosphotransferase II (*nptII*) driven by nopaline synthase promoter was the plant selectable marker gene. A single colony of *Agrobacterium* strain EHA 105/pBinAR was picked from a fresh plate and grown overnight at 28°C in LB medium (pH 7.0) with 50 mg/L kanamycin and 10 mg/L rifampicin. The bacterial culture was later re-suspended in 100 mL of Winans’ AB medium (pH 5.2) and incubated for 18 h at 28°C. Two-day old seedlings were used for *in planta* transformation using the standardized protocol (Karthik et al., 2020a; Kesiraju et al., 2020). The primary transformants were transferred to the greenhouse and allowed to set seeds.

### Selection Agent-Based Screening for the Identification of Putative Transformants in T_1_ Generation

Seeds of primary transformants independently harbouring *cry1AcF* and *cry2Aa* were harvested and screened for the identification of putative transformants. Initially, standardization of kanamycin concentration for screening was carried out in the wild type seedlings. For this, kanamycin solution of different concentrations (10, 25, 50, 70, 90, 100, 125, 150, 175, 200, 225, 250, 275, and 300 mg/L) were prepared in 100 mL of distilled water. Thirty seedlings of wild type (cv. P8–6) were dropped into each of the solutions and incubated at 50 rpm for 5 h. The seedlings were later transferred to soilrite, maintained under net house conditions for 7–10 days and observed for necrosis. Seeds treated with water were grown separately and labelled as untreated wild type.

For the identification of putative transformants, 30 seeds from each of the primary transformants and wild type were treated with 150 mg/L of kanamycin for 5 h and allowed to grow under net house conditions. Wild type plants treated with water (untreated wild type) and kanamycin (treated wild type) were also maintained separately. The seedlings with well-established shoots and roots were later transferred to pots and further analysed for transgene integration, expression and insecticidal efficacy. The identity of the transgenic plants was based on numbering with the *cry1AcF* starting with 1- and 2- for *cry2Aa* transgenic plants while the subsequent numbers designated the generation viz., T_0_–T_1_–T_2_ etc.

### Molecular Analyses for T-DNA Integration

#### Genomic DNA Isolation

Topmost leaves of transgenic and wild type plants of cotton were freshly collected in liquid nitrogen. Genomic DNA was isolated following a modified cetyl trimethyl ammonium bromide (CTAB) method for plants with high polysaccharides and poly phenols (Porebski et al., 1997).

##### Polymerase chain reaction analysis of transformants

Polymerase chain reaction (PCR) analysis of transformants in different generations was performed to amplify target genes *cry1AcF* and *cry2Aa* as well as the marker gene (*nptII* gene) using various primers. In T3 generation, PCR analysis was performed with *nptII* gene and T-DNA specific right border primers (Table 1). Each PCR reaction (25 μL) consisted of 100 ng genomic DNA, 2.5 μL of 10 × Taq buffer (10 mM pH 9.0 Tris–HCl, 50 mM KCl, 1.5 mM MgCl_2_, 0.01% gelatin), 10 pM each of forward and reverse primers, 200 μM dNTPs, and 1 U of Taq DNA polymerase (Bangalore Genei, Bengaluru, India); volume made upto 25 μL with nuclease-free water. “Blank” contained nuclease-free water instead of genomic DNA, negative control contained 100 ng of genomic DNA from wild type and positive control contained 25 ng of the binary vector. PCR amplification was carried out in a thermal cycler (Eppendorf, Hamburg, Germany) programmed with an initial denaturation step at 95°C for 5 min followed by 35 cycles of denaturation at 95°C for 1 min, annealing at 58°C for 1 min, extension at 72°C for 1 min (for *nptII*, T-DNA specific right border and *cry 2Aa* gene) and 1 min 30 s for *cry* 1*AcF* gene and a final extension at 72°C for 10 min. The amplified products were visualized by electrophoresis on a 0.8% agarose gel.

**TABLE 1:** List of primers used in the study.

Primer ID	Primer sequence (5′–3′)	Size of amplicons (kb)
*nptII* FP	CCGGAATTCATGATTGAACAA	750 bp
*nptII* RP	CCCAAGCTTCAGAAGAACTC	
*cry1AcF* FP	AACCCAAACATCAACGAGTGC	1.8 kb
*cry1AcF* RP	CGTACAAGTGGAGGACCCTTTGC	
*cry2Aa* FP	TCAGGGACGTGATCCTCAACGC	1 kb
*cry2Aa* RP	TCGCCCTGGTTGCCGAACTT	
T-DNA right border FP	ATTGGCGGGTAAACCTAAGAG	250 bp
T-DNA right border RP	CTGTATGCGTTGGTGCAATTT	

##### Genomic southern analysis

To perform Southern hybridization, 15 μg of genomic DNA (transgenic and wild type plants) was digested with *Hin*dIII (NEB high fidelity, NEB). The digested DNA samples were then separated by electrophoresis on a 0.8% agarose gel and transferred on to a positively charged nylon membrane (Bio-Rad, Hercules, CA, United States) by capillary blotting. A 750 bp *nptII* gene fragment was labelled with DIG PCR labelling kit (Roche Holding AG, Basel, Switzerland) and used as probe for hybridization. Hybridization, washing and development were carried according to manufacturer’s instructions (Roche Holding AG, Basel, Switzerland).

#### Analysis of Protein Expression in Cotton Transformants Harbouring *cry1AcF* and *cry2Aa* Genes

##### Isolation of total proteins

Total proteins were isolated from 100 mg leaf samples of both transgenic and wild type plants by finely grinding with liquid nitrogen and later reconstituting in 500 μL of extraction buffer [0.1 M Tris-HCl, 0.5 M EDTA, 30% sucrose, 1% PVPP, 0.1 M KCl, 2% sodium dodecyl sulphate (SDS), 1 mM phenylmethane sulfonyl fluoride, 5% 2-mercaptoethanol; pH8.8] (Wu et al., 2014). The isolated proteins were quantified by Bradford’s assay (Bio-Rad, Hercules, CA, United States).

##### Western blot analysis

For western blot analysis, total proteins (20–25 μg) were denatured, subjected to SDS–PAGE (sodium dodecyl sulfate–polyacrylamide gel electrophoresis) and blotted onto nitrocellulose membrane (Millipore, Burlington, MA, United States) at a constant voltage (40 V) for 3 h in transfer buffer (1.5 g Tris, 7.2 g glycine and 100 mL methanol made up to 500 mL with distilled water). Development of blots was initiated by blocking using the NAP (non-animal protein) blocker (G-Biosciences, St. Louis, MO, United States) followed by an initial hybridization with 1:3,000 dilution of cry protein-specific primary antibody (Amar Immunodiagnostics, Jubilee Hills, Hyderabad, India) and subsequently with 1:6,000 dilution of HRP (horseradish peroxidise) conjugated secondary antibody (Bangalore Genei, Bengaluru, India). The blots were washed four times with 1X PBST (phosphate-buffered saline) followed by the addition of TMB (3,3′,5,5′-Tetramethylbenzidine) substrate (Promega, Madison, WI, United States) for colour development.

##### Expression analysis by ELISA

Cry protein expression in transgenic cotton events was carried out with three biological and three technical replicates using commercially available ELISA plates pre-coated with specific antibodies according to the manufacturer’s instructions (Amar Diagnostics, Hyderabad, India). The amount of protein in transgenic events was measured, average values were plotted and comparison between events was done by one-tailed *T*-test with significance of *p* = 0.05.

#### Efficacy Analysis of Cotton Transformants Independently Expressing *cry1acf* and *cry2aa* Genes Against *Helicoverpa armigera* Challenge

*Helicoverpa armigera* larvae were collected from IARI (Indian Agricultural Research Institute, New Delhi) chickpea fields during March, raised on artificial diet under growth conditions of 26 ± 1°C, 70–80% relative humidity and 16 h light–8 h dark. Male and female adults were fed with 10% sucrose solution and maintained under specified growth conditions. Eggs were collected, allowed to hatch and larvae were reared on artificial diet. All the larvae were maintained together till second instar and were later separated to avoid cannibalism.

Efficacy of cotton transgenic plants *vis-à-vis* wild type plants against *H. armigera* was assessed in leaves by conducting *in vitro* bioassays with transgenic plants of T_1_, T_2_, and T_3_ generations. Bioassays were conducted with 45–55 days old plants in all the generations. Fully expanded third and fourth leaves were excised and the petiole was covered with wet cotton to maintain moisture in the Petri plates. Two technical replicates from each plant were maintained for each treatment and ten neonate larvae were released onto each leaf. Larval mortality and extent of damage on the plant tissue were recorded at 24, 48, 72, and 96 h after release of larvae. The percent larval mortality was calculated by counting the number of dead larvae and percent leaf damage was calculated by the area of leaf damaged by the feeding larvae (Rathinam et al., 2021). Bioassay of selected transformants by challenging bolls with a single fourth instar larva of *H. armigera* was performed in T_3_ generation. The ability to resist the larval challenge was assessed for 96 h as in the leaf bioassay.

Bioassay with T_1_ generation plants was performed with individual plants in two technical replicates and the average was presented. Efficacy analysis in advanced generations was performed with a larger number of biological replicates by taking two technical replicates for each biological replicate. The resultant T_2_ and T_3_ bioefficacy data was subjected to statistical analysis. Analysis of variance (ANOVA) was calculated followed by mean separation by the Student–Newman–Keuls’ test (*p* = 0.05). Graphs and regression models were generated using R statistical language version 3.4.3 (2019-2107-30). Tukey’s honestly significant difference (HSD) test was performed with T_3_ generation events to assess significant variance between means revealed by ANOVA.

## Results and Discussion

Use of biotechnological approaches for the deployment of transgenes into agronomically important crops like cotton have been the most successful and widely adopted technology worldwide. Transgenic cotton resistant to boll worm has been one of the commendable contributions made by the scientific fraternity toward agricultural sustainability. However, introgression of genes for multiple pest mitigation and management of resistance development in insects are important. Thus the present study is an effort to demonstrate the efficacy of two novel *Bt* genes independently in transgenic cotton for pest management.

### Development of Transgenic Cotton Plants

Owing to the recalcitrance of cotton to regeneration, transgenic plants were generated by targeting the desired genes individually to the shoot apical meristem following an *in planta* transformation strategy (Karthik et al., 2020a; Kesiraju et al., 2020). A total of 4,600 seedlings of cotton cultivar P8–6 were subjected to *Agrobacterium* transformation independently for *cry1AcF* and *cry2Aa*. Nearly 5% of plants recovered from the infection stress, were transferred to pots and maintained under net house conditions. Consequently, 95 plants harbouring *cry1AcF* gene and 82 plants harbouring *cry2Aa* gene could establish, produce bolls with seeds. Since the T_0_ plants produced are chimeric in nature, the putative transformants were identified in the T_1_ generation (Kesiraju et al., 2020).

This strategy of *in planta* transformation has been utilized for the successful transformation of different crop species (Kesiraju and Sreevathsa, 2017); including fiber crops like cotton (Karthik et al., 2020a; Kesiraju et al., 2020) and flax (Karthik et al., 2020b). Moreover, development of transgenic cotton resistant to *H. armigera* has been a pertinent endeavour by various research groups (Tabashnik et al., 2002; Wu et al., 2003; Kurtz et al., 2007; Anilkumar et al., 2008; Singh et al., 2016; Han et al., 2017; Bajwa et al., 2020; Jadhav et al., 2020; Katta et al., 2020).

### Screening Under Kanamycin Selection Pressure for the Identification of Putative Transformants

The major advantage offered by tissue culture-independent transformation strategies has been the ability to develop a large number of primary transformants. This demands the development of stringent screening methodologies for the identification of putative transformants in the subsequent T_1_ generation. Screening is carried out in the presence of selection agents based on the selectable marker genes in the binary vector.

**FIGURE 1:**
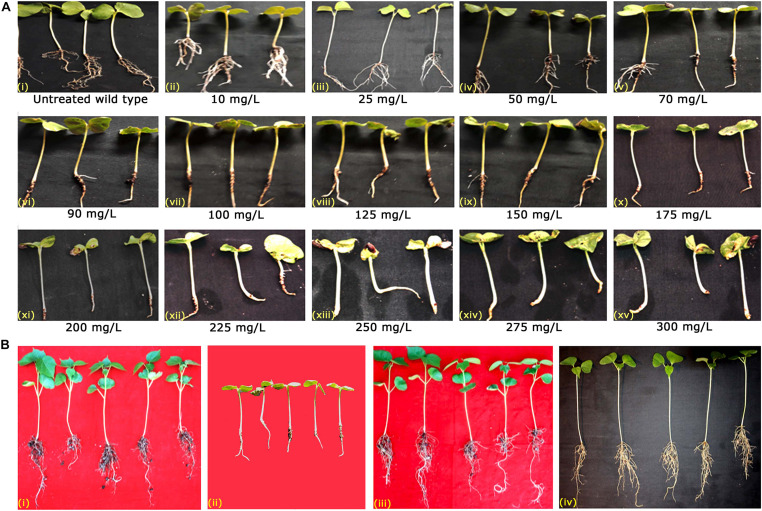
Kanamycin screening for the identification of putative transformants. **(A)** Standardization of kanamycin concentration in cv. P8–6. Variation in the response of wild type seedlings to different kanamycin concentrations **(i)** untreated wild type, **(ii)** 10 mg/L, **(iii)** 25 mg/L, **(iv)** 50 mg/L, **(v)** 70 mg/L, **(vi)** 90 mg/L, **(vii)** 100 mg/L, **(viii)** 125 mg/L, **(ix)** 150 mg/L, **(x)** 175 mg/L, **(xi)** 200 mg/L, **(xii)** 225 mg/L **(xiii)** 250 mg/L, **(xiv)** 275 mg/L, and **(xv)** 300 mg/L. **(B)** Response of T_1_ generation cotton seedlings to 150 mg/L of kanamycin **(i)** untreated wild type, **(ii)** treated wild type, and **(iii,iv)** Kanamycin-resistant T_1_ generation transgenic plants.

In the present study, as pBinAR vectors with genes *cry1AcF* and *cry2Aa* possessed *nptII* gene as the plant selectable marker, putative transformants were selected in the presence of the antibiotic kanamycin. The concentration of kanamycin deleterious to cotton cv. P8–6 was initially assessed in the wild type seedlings and scored for necrosis. Seedlings in the presence of 10–70 mg/L kanamycin showed no symptoms of necrosis with healthy roots (Figures 1Aii–v). However, seedlings treated with kanamycin concentrations of 90 mg/L and above exhibited symptoms of necrosis. Retardation in root growth was observed with further raise in kanamycin concentration (Figures 1Avi–xi). Further, severe growth retardation was observed in plants treated with >225 mg/L kanamycin (Figures 1Axii–xv) as evidenced by necrotic leaves, poor root development and reduced height of plants. While plants treated with water (untreated wild type) remained healthy (Figure 1Ai).

Based on the standardization of kanamycin concentration in the wild type seedlings, 150 mg/L kanamycin was used to screen T_1_ generation seedlings. About 390 seeds of primary transformants of cotton with *cry1AcF* gene and 180 with *cry2Aa* were subjected to kanamycin treatment and planted in trays containing soilrite. Among them, 65 plants of *cry1AcF* (16.6% of plants) and 33 plants of *cry2Aa* (18.3% of plants) could resist kanamycin induced selection pressure (Figures 1Biii,iv) and exhibited robust phenotype on par with the untreated wild type (Figure 1Bi). The remaining plants exhibited necrotic symptoms as in the case of treated wild type (Figure 1Bii) and were discarded. Recovered plants were later transferred to pots filled with soil and allowed for growth under nethouse conditions.

The selection agent-based preliminary analysis for the identification of putative transformants allows in effective segregation of the pool of T_1_ generation plants and identification of high expressing transformants due to the high selection pressure used. Identification of putative transformants resistant to kanamycin also reiterated the amenability of cotton cultivar P8–6 to *in planta* transformation. The authenticity of soilrite-based seedling level screening was successfully demonstrated earlier with glyphosate (Karthik et al., 2020a) and hygromycin (Kesiraju et al., 2020) in the same cotton genotype for the identification of putative transformants.

Based on the selection of T_1_ generation plants in the presence of 150 mg/L kanamycin, 65 and 33 plants harbouring *cry1AcF* and *cry2Aa*, respectively, were selected for further molecular analysis to demonstrate T-DNA integration.

### Molecular Characterisation of T_1_ Generation Transgenic Plants With *cry1AcF* Gene

**FIGURE 2:**
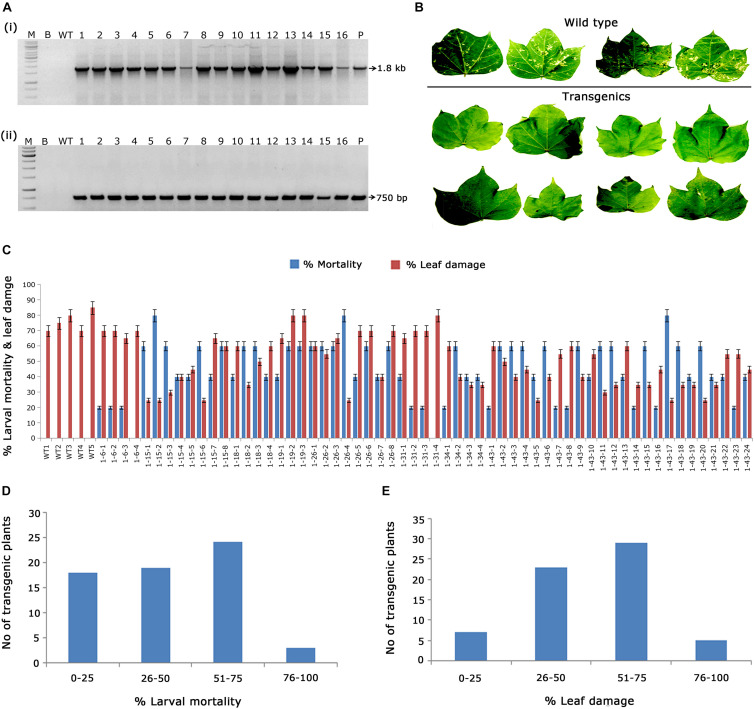
Molecular analysis of *cry1AcF* cotton transformants in T_1_ generation. **(A)** Polymerase chain reaction (PCR) analysis of T_1_ generation *cry1AcF* plants for the amplification of **(i)** 1.8 Kb *cry1AcF* gene and **(ii)** 750 bp *nptII* gene. Lane M – 1 Kb Ladder (Thermo Scientific), Lane B – water blank (all PCR components without template DNA), Lane WT – wild type (100 ng), and Lanes 1–16: T_1_ generation transgenic plant DNA (100 ng). Lane P – positive control pBinAR *cry1AcF* (25 ng). **(B)** Representative leaves of wild type and transgenic plants challenged with *Helicoverpa* armigera in a detached leaf bioassay. **(C)** Graphical representation of the performance of 59 transgenic plants and wild type in terms of percentage of larval mortality and leaf damage. **(D,E)** Overview of the efficacy of transgenic plants against deliberate challenging of *H.*armigera larvae.

As a preliminary analysis, PCR amplification of 1.8 kb c*ry1AcF* and 750 bp *nptII* gene fragments in kanamycin-resistant transgenic plants confirmed the presence of T-DNA (Figures 2Ai,ii). Furthermore, evaluation of transgenics in terms of their efficacy toward the target pest forms the bottom line of the study. Short listing of events across generations was mainly dependent on their performance in the *in vitro* bioassays against deliberate *H. armigera* challenge. Accordingly, 59 T_1_ generation plants with *cry1AcF* were examined for their efficacy against *H. armigera* (Figure 2B). The efficiency of transgenic cotton plants in terms of larval mortality and leaf damage (Figure 2C) showed that nearly 25 plants of *cry1AcF* exhibited larval mortality greater than 60% and controlled leaf damage of <50% (Figures 2D,E).

#### Molecular Characterisation of T_1_ Generation *cry2Aa* Transgenic Plants

**FIGURE 3:**
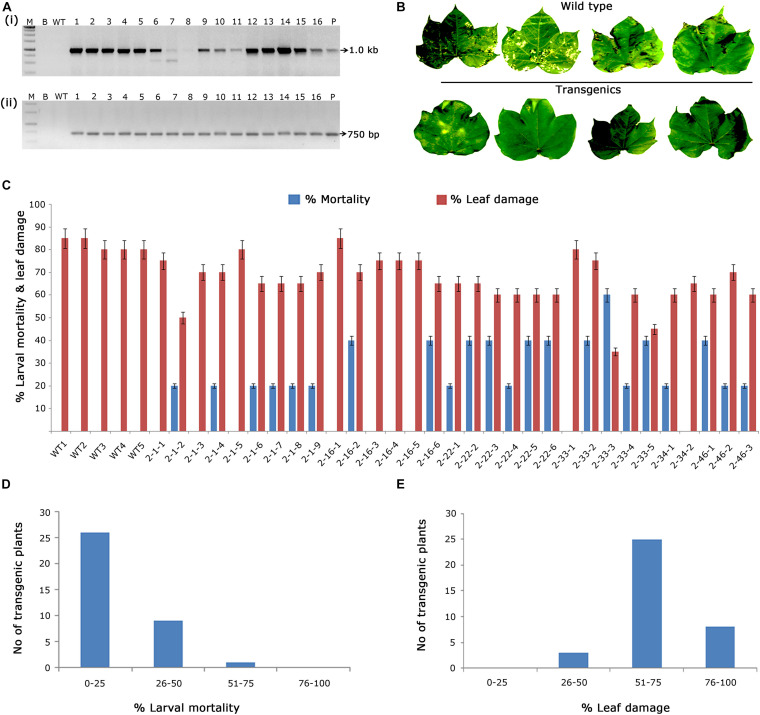
Molecular analysis of *cry2Aa* cotton transformants in T_1_ generation. **(A)** PCR analysis of T_1_ generation *cry2Aa* plants for the amplification of **(i)** 1.0 Kb *cry2Aa* gene; **(ii)** 750 bp *nptII* gene. Lane M – 1 Kb Ladder (Thermo Scientific), Lane B – water blank (all PCR components without template DNA), Lane WT – wild type (100 ng), and Lanes 1–16: T_1_ generation transgenic plant DNA 100 ng. Lane P – positive control pBinAR *cry2Aa* (25 ng). **(B)** Representative leaves of wild type and transgenic plants challenged in insect leaf bioassay. **(C)** Graphical representation of the performance of 30 transgenic plants and wild type in terms of percentage of larval mortality and leaf damage. **(D,E)** Overview of the efficacy of transgenic plants against deliberate challenging of *Helicoverpa* armigera larvae.

In the case of putative transformants harbouring *cry2Aa*, PCR amplification of 1.0 kb c*ry2Aa* and 750 bp *nptII* gene fragments in transgenic plants and absence in wild type plants (Figures 3Ai,ii) indicated the presence of T-DNA. Accordingly, 30 PCR positive plants were challenged with *H. armigera* to determine their efficacy (Figures 3B,C), out of which, three plants exhibited larval mortality between 50 and 75% and leaf damage of < 50% (Figures 3D,E). However, wild type plants showed leaf damage ranging between 70 and 90%.

Based on the two levels of assessment i.e., screening under kanamycin selection pressure and efficacy toward *H. armigera*, 6.4% of *cry1AcF* and 1.6% of *cry2Aa* plants were identified as promising. Though kanamycin-selected transformants established in a slower pace compared to wild type plants, the seed pool obtained from these plants were normal and viable. The seeds of these plants were collected and advanced for characterization in T_2_ generation.

### Characterisation of T_2_ Generation Transgenic Plants With *cry1AcF* and *cry2Aa* for T-DNA Inheritance and Expression

In the case of *cry1AcF*, 17 events out of 25 and 3 selected events of *cry2Aa* that exhibited >60% larval mortality and <30% leaf damage were advanced to T_2_ generation. For inheritance and integration analyses, 10 seeds of each of the selected events of *cry1AcF* and 20 seeds of each selected event with *cry2Aa* were germinated and planted in pots filled with soil and grown under net house conditions.

**FIGURE 4:**
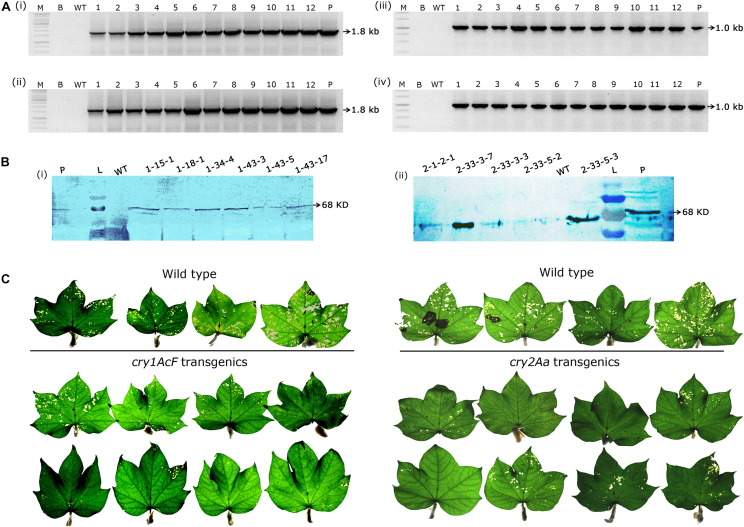
Molecular analysis of transformants in T_2_ generation. **(A)** PCR analysis of T_2_ generation progeny plants for the amplification of **(i,ii)** 1.8 Kb *cry1AcF* gene and **(iii,iv)** 1.0 Kb *cry2Aa* gene. Lane M – 1 Kb Ladder (Thermo Scientific), Lane B – water blank (all PCR components without template DNA), Lane WT– wild type (100 ng), and Lanes 1–12 of **(i,ii)** two progeny plant of events–1-15-1, 1-18-1, 1-18-3, 19-1, 1-26-1, 1-26-2, 1-34-2, 1-34-3, 1-34-4, 1-43-3, 1-43-5, and 1-43-17, and Lanes 1–12 of **(iii,iv)** eight progeny plant of events–2-1-2, 2-33-3, and 2-33-5. Lane P–positive control (binary vector) pBinAR *cry1AcF* and *cry2Aa*, respectively. **(B)** Western blot analysis of selected transgenic plants harboring **(i)** *cry1AcF* and **(ii)** *cry2Aa*. Lane L – pre-stained protein ladder, Lane WT – wild type, Lanes L – pre-stained protein ladder, transgenic plants, and Lane P – purified protein of *cry1AcF* and *cry2Aa* (30 ng). **(C)** Assessment of the performance of wild type and transgenic plants in detached leaf bioassay challenged with *Helicoverpa* armigera.

Genomic PCR analysis demonstrated the stable inheritance of the integrated transgene in selected events and the desired amplicons of 1.8 kb *cry1AcF* (Figures 4Ai,ii) and 1.0 kb *cry2Aa* (Figures 4Aiii,iv) gene fragments were obtained in the respective plants. Further, western blot analysis provided evidence for transgene expression at protein level (Figures 4Bi,ii) as both sets of transgenic plants developed a single intense band of 68 KDa at the xpected position against their respective antibodies. However, the desired band was absent in the wild type.

In the present study, the major focus was toward the identification of promising transgenic cotton events with evidences for stable integration, inheritance and superiority in their efficacy against *H. armigera*. Unequivocal demonstration of the efficacy of T_2_ generation transgenic events against boll worm challenge was provided by the detached leaf bioassay. Leaves of 7–8 *cry1AcF* progeny plants of each of the 17 events and 15 *cry2Aa* progeny plants of each of the three events were taken in two replicates and challenged with 10 neonate larvae of *H. armigera.* Accordingly, a total of 128 plants of *cry1AcF* and 44 plants of *cry2Aa* were subjected to *in vitro* bioefficacy analysis in T_2_ generation. The incurred leaf damage in the wild-type *vis a vis* transgenics of *cry1AcF* and *cry2Aa* was analysed (Figure 4C).

**FIGURE 5:**
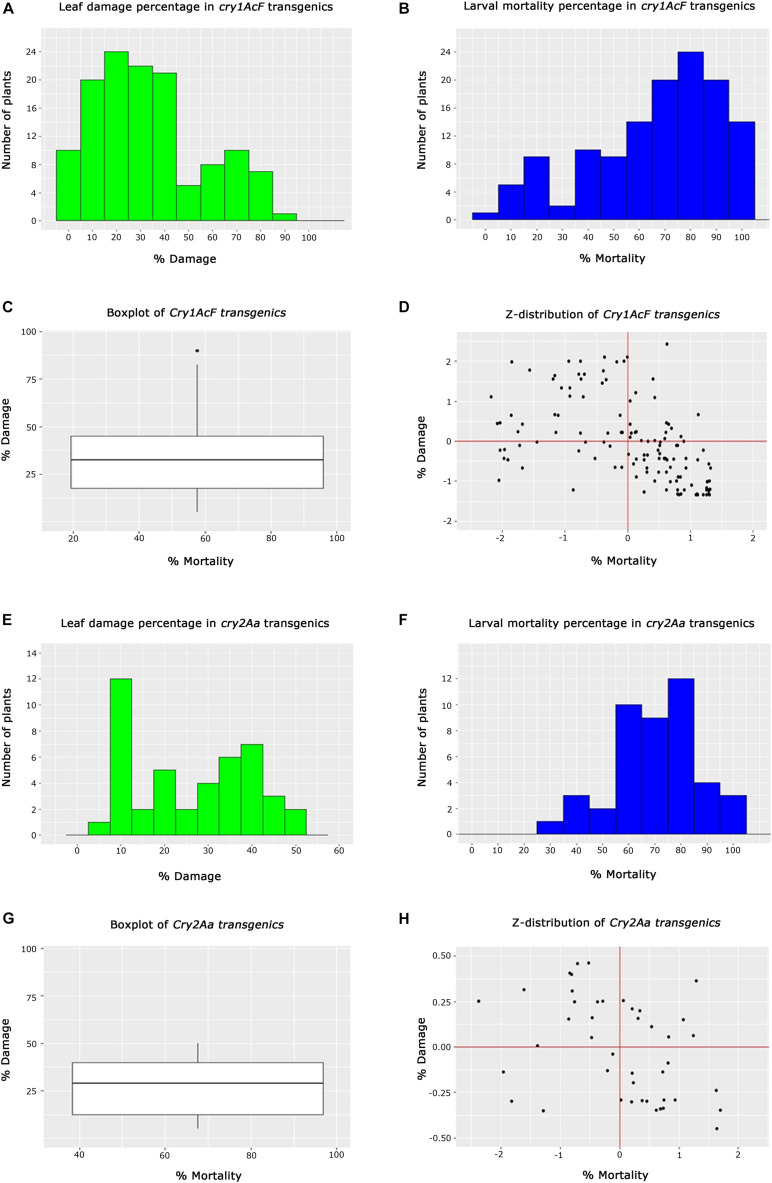
Analysis of T_2_ generation transgenic cotton plants toward *Helicoverpa* armigera challenge. **(A,B)** Histograms representing the performance overview of *cry1AcF* transgenic plants based on % leaf damage and % larval mortality, respectively, **(C)** box whisker plot depicting the performance spread of *cry1AcF* transgenic plants, **(D)** Z-distribution analysis of *cry1AcF* transgenic plants; **(E,F)** Histograms representing the overview of *cry2Aa* transgenic plants based on % leaf damage and % larval mortality, respectively, **(G)** box whisker plot depicting the performance spread of *cry2Aa* transgenic plants, and **(H)** Z-distribution analysis of *cry2Aa* transgenic plants.

In the case of *cry1AcF* transgenics, 10 plants out of 128 belonging to different events exhibited <5% leaf damage, while 44 plants showed <25%. Another set of 43 plants exhibited leaf damage ranging between 26 and 45%. However, 31 plants depicted a greater percentage of leaf damage ranging between 46 and 95% (Figure 5A). This showed that the performance of all the selected transgenic plants was not identical in resisting the insect herbivory. Similarly, an overview of larval mortality identified 27 plants with <45% larval mortality while 67 plants exhibited mortality between 46 and 85%. Alternatively, 34 plants showed maximum mortality of 86–100% (Figure 5B). Box whisker plots showed that large number of plants showed larval mortality of >58% and leaf damage of less than 38% in *cry1AcF* plants (Figure 5C). Z-distribution analysis identified 11 events of *cry1AcF* in the first quadrant for larval mortality and leaf damage (Figure 5D).

In the case of *cry2Aa* events, efficacy analysis of 44 plants toward bollworm challenge showed 20 plants to be exhibiting leaf damage ranging between 5 and 20%; 19 plants showed leaf damage ranging between 22 and 42% and 5 plants with leaf damage between 45 and 55% (Figure 5E). The performance of these plants in terms of larval mortality depicted that five plants showed low larval mortality between 25 and 55%, 32 plants exhibited efficient larval mortality ranging between 56 and 85% and seven plants exhibited maximum mortality between 86 and 100% (Figure 5F). Box whisker plot of *cry2Aa* plants, plants showed larval mortality >68% and leaf damage less than 30% (Figure 5G). Z-distribution analysis identified few plants of three *cry2Aa* events in the first quadrant for the two parameters (Figure 5H).

**FIGURE 6:**
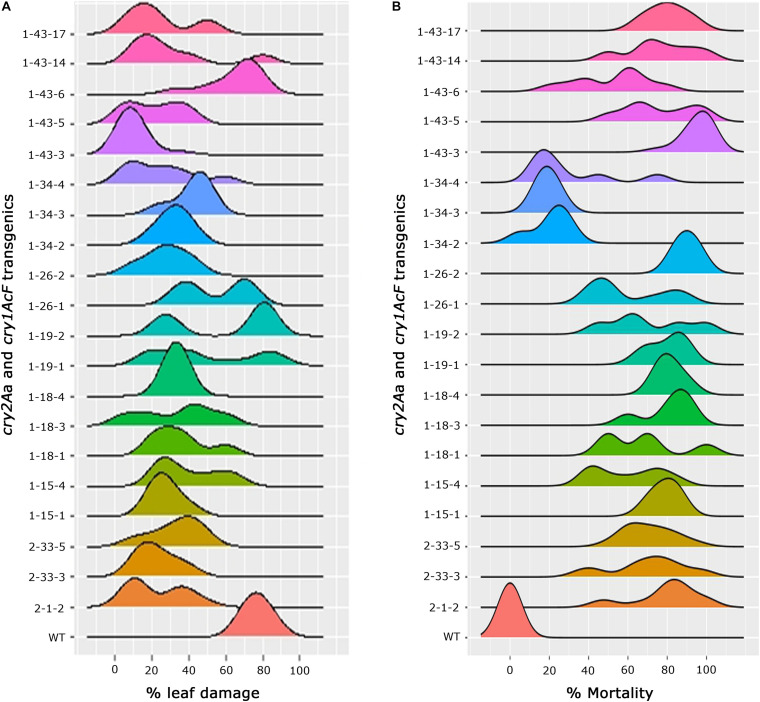
Overview of efficacy analysis of T_2_ generation progeny plants of cotton transformants harbouring *cry1AcF* and *cry2Aa* **(A)** percentage of leaf damage and **(B)** percentage of larval mortality.

In-depth analysis of the overall performance of the progeny plants across various events was attempted to not only assess the stability of bioefficacy but also to narrow down on the promising events (Figures 6A,B). Comprehensive overview of the ability of transgenic plants to resist bollworm challenge emerged out of the analysis. Accordingly, explicit susceptibility to *H. armigera* was showcased by the wild type plants which showed increased leaf damage with no larval mortality. Conversely, the selected events of cotton harbouring *cry1AcF* and *cry2Aa* though effective toward the insect challenge, varied across the progeny. While the progeny of some of the events were uniform in their performance in the bioassay, there was variation in some (Figures 6A,B). A single peak was observed in a group of events exhibiting consistent leaf damage with medians ranging between 10 and 30% which included events, 2-1-2, 2-33-3, and 2-33-5 of *cry2Aa*; 1-15-1, 1-18-1, 1-18-3, 1-18-4, 1-19-1, 1-26-2, 1-34-2, 1-34-3, 1-34-4, 1-43-3, 1-43-5, 1-43-14, and 1-43-17 of *cry1AcF*. In the remaining events, two peaks were observed indicating mixed performance of the progeny plants and exhibiting medians ranging between 20 and 60% (Figure 6A). Similarly, median values for percent larval mortality ranged between 65 and 90% in events 2-1-2, 2-33-3, and 2-33-5 of *cry2Aa*; 1-15-1, 1-18-1, 1-18-3, 1-18-4, 1-19-1, 1-26-2, 1-43-3, 1-43-5, 1-43-14, and 1-43-17 of *cry1AcF* (Figure 6B).

Accordingly, on the basis of statistical analysis and its correlation with the performance of larvae during the bioassay led us closer to superior events. For instance, in the case of events, 1-18-1 and 1-34-4, mortality of maximum number of larvae occurred in the initial phases of the bioassay, but few larvae stopped feeding became week and died during 72–96 h time period and were thus selected.

Consequently, 13 events (2-1-2, 2-33-3, and 2-33-5 of *cry2Aa*; 1-15-1, 1-18-1, 1-18-3, 1-19-1, 1-26-2, 1-34-2, 1-34-4, 1-43-3, 1-43-5, and 1-43-17 of *cry1AcF*) exhibiting maximum larval mortality of 80–90% and leaf damage of 20–30% were shortlisted and identified to be superior in terms of their performance. The seeds of all these plants were harvested individually for raising T_3_ plants.

### Assessment of Cotton Transformants Harbouring *cry1AcF* and *cry2Aa* in the Advanced T_3_ Generation

**FIGURE 7:**
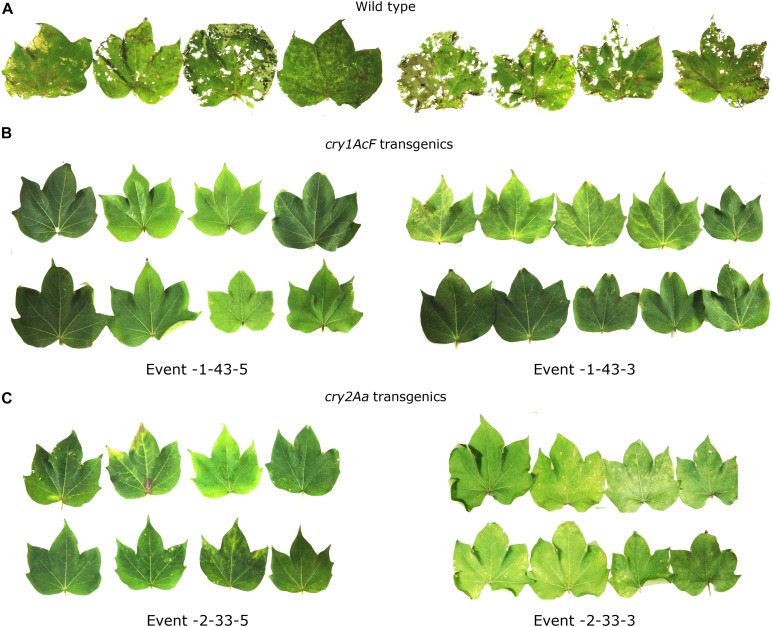
*In vitro* detached leaf bioassay to demonstrate stability of the selected transgenic events to boll worm challenge in T_3_ generation. Leaves of **(A)** wild type and **(B,C)** transgenic plants of *cry1AcF* and *cry2Aa* events challenged with 10 neonate larvae of *Helicoverpa* armigera.

The selected 13 transgenic events were subjected to various molecular and bioefficacy characterization for the assessment of stable integration and inheritance of the T-DNA. Stability in the bioefficacy analysis was considered as the primary evaluation step for the analysis of the transformants (Figures 7A–C). Highly regulated leaf damage was observed in replicates of maximum number of plants within the events showing the stability of the transgenes in the progeny plants.

**FIGURE 8:**
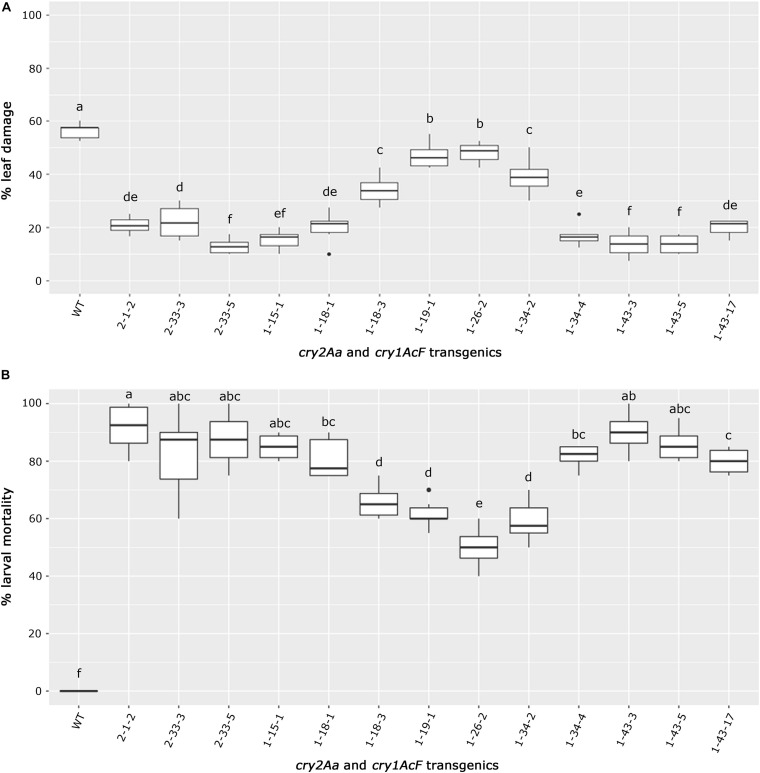
Overview of bioefficacy analysis of transgenic plants in T_3_ generation. Box plots representing **(A)** leaf damage, and **(B)** larval mortality in selected *cry2Aa* and *cry1AcF* events and their respective wild type plants. The significant differences among the transgenic events and wild types were analyzed by Tukey’s HSD test with *p* < 0.05.

The selected transgenic events could be categorized based on their performance in the bioassay (Figures 8A,B). Best performing events 1-43-3, 1-43-5, and 1-15-1 of *cry1AcF* and 2-33-5 of *cry2Aa* emerged as a group due to least leaf damage that varied between 10 and 15%. Further, events exhibiting a little higher leaf damage of 10–20% (events 2-1-2 of *cry2Aa* and 1-18-1, 1-43-17 of *cry1AcF*) formed another group. However, event 2-33-3 and 1-34-3 were grouped separately due to 20–30% leaf damage. The remaining transgenic plants exhibiting >30% leaf damage and wild type with 60% leaf damage emerged as distinct groups (Figure 8A).

Similarly, transgenic events 2-1-2, 2-33-3, and 2-33-5 of *cry2Aa*; 1-15-1, 1-18-1, 1-34-4, 1-43-3, 1-43-5, and 1-43-17 of *cry1AcF* could be categorized as a promising group with larval mortality ranging between 70 and 100%. Remaining transgenic events exhibiting larval mortality between 50 and 70% and wild type with null larval mortality were distinctly separate (Figure 8B).

**FIGURE 9:**
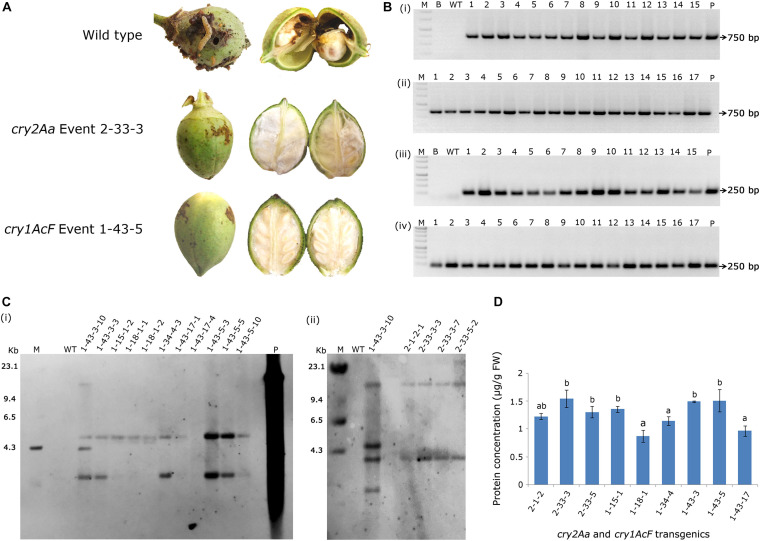
Assessment of bolls of cotton transformants to *Helicoverpa* armigera challenge and molecular characterization of superior transgenic events. **(A)** Representative bolls of wild type and transgenic events, and **(B)** PCR analysis of T_3_ generation progeny plants for the amplification of **(i,ii)** 750 bp *nptII* gene and **(iii,iv)** 250 bp T-DNA right border specific fragments. Lane M – 1 Kb Ladder (Thermo Scientific), Lane B – Water Blank (all PCR components but without template DNA), Lane WT – wild type (100 ng), Lanes 1–15 of **(i,iii)** and Lanes 1–17 of **(ii,iv)** represents: four progeny plant of events–2-1-2, 2-33-3, 2-33-5, 1-15-1, 1-18-1, 1-34-4, 1-43-3, and 1-43-5. Lane P–positive control (binary vector) pBinAR *cry1AcF*. **(C)** Genomic Southern analysis of transgenic plants probed with 750 bp DIG-labelled *nptII* gene **(i)** transgenic cotton plants with *cry1AcF* and **(ii)** transgenic cotton plants with *cry2Aa*. Lane M – Lambda *Hin*dIII DNA ladder, Lane P – linearized plasmid pBinAR *cry1AcF* (10 pg), Lane WT – wild type, transgenic plants. **(D)** ELISA for cry protein (μg/g FW) expression analysis in leaf tissues of different transgenic events.

As a result, nine events (2-1-2, 2-33-3, and 2-33-5 of *cry2Aa*; 1-15-1, 1-18-1, 1-34-4, 1-43-3, 1-43-5, and 1-43-17 of *cry1AcF*) that exhibited larval mortality ranging between 70 and 100% and leaf damage of <30% emerged as superior (Figures 8A,B). Additionally, challenging bolls of two promising *cry1AcF* and *cry2Aa* transgenic plants with a single fourth instar *H. armigera* larva reiterated their superiority (Figure 9A). While bolls of the wild type plants showed maximum damage due to voracious feeding of *H. armigera* larvae, bolls of the transgenic plants could not be damaged by the attacking insect (Figure 9A). Though the larva initiated feeding, penetration in the bolls of transgenic plants could not be achieved.

In T_3_ generation, molecular analysis of the selected events by PCR confirmed the presence of *nptII* gene and T-DNA specific right border in the progeny of transgenic plants with both the genes indicating integration of the complete T-DNA. Genomic DNA of the wild type plants did not show any amplification (Figures 9Bi–iv). Further, genomic Southern analysis precisely identified the independent nature of the transformants. Probing the blots with a 750 bp DIG-labelled *ntpII* gene fragment showed a two copy integration in most of the events (1-34-4-3, 1-43-3-3, 1-43-17-1, 1-43-17-4, 1-43-5-3, 1-43-5-5, and 1-43-5-8 of *cry1AcF*; 2-1-2-1, 2-33-3-3, 2-33-3-7, and 2-33-5-2 of *cry2Aa*); single copy integration in events 1-15-1-2 and 1-18-1-1 of *cry1AcF*, while four copies of the T-DNA was found in the event 1-43-3-10 of *cry1AcF* (Figures 9Ci,ii). No hybridisation signal was observed in wild type plants. In an effort to correlate T-DNA integration and bioefficacy analysis, *cry1AcF* and *cry2Aa* proteins were quantified by ELISA in the respective transformants (Figure 9D). It was observed that the protein expression was in the range of 1.0–1.5 μg/g FW in the selected transformants. Despite the fact that the level of proteins expressed varied across studies when compared to the *cry* genes used in the present study, efficacy against the pest was nevertheless demonstrated.

The study therefore is yet another proof to ascertain that amalgamation of various tools and strategies is required for the effective control of *H. armigera*. In accordance to our previous demonstration, ∼2% of promising cotton transformants could be identified using the methodology. Improved performance of the overall population of T_2_ and T_3_ generation plants toward resisting *H. armigera* supported the stringent molecular and bioefficacy exploited in the study. The study thus delineates the usefulness of two *Bt* ICPs under study in the mitigation of *H. armigera.* Further, the amenability of cotton to the non-tissue culture based approach and development of a large number of transformants is reassured. The set of promising events identified in the study can be utilized in crop improvement programmes of cotton for the management of boll worm.

## Data Availability Statement

The raw data supporting the conclusions of this article will be made available by the authors, without undue reservation.

## Author Contributions

KK developed the transgenic plants, performed bio efficacy, molecular analyses, and wrote and prepared the manuscript. JN performed the statistical analysis. MR helped in preparing the manuscript. NS was responsible for data analysis and critical editing of the manuscript. RS is responsible for acquisition of funds, designing experiments, critical editing, and revising the manuscript. All authors contributed to the article and approved the submitted version.

## Conflict of Interest

The authors declare that the research was conducted in the absence of any commercial or financial relationships that could be construed as a potential conflict of interest.

## Footnotes

1 https://cotcorp.org.in/national_cotton.aspx
